# Effect of Ultraviolet Photofunctionalization of Sandblasted and Acid-Etched Titanium Surfaces on Dental Pulp Stem Cell Behavior: An In Vitro Study

**DOI:** 10.7759/cureus.109773

**Published:** 2026-05-27

**Authors:** Kranti Chavare, Yogesh Khadtare, Vidya Dodwad, Pooja Pharne, Santosh B Jadhav, Sanpreet S Sachdev

**Affiliations:** 1 Department of Periodontology, Bharati Vidyapeeth (Deemed to Be University) Dental College and Hospital, Pune, IND; 2 Department of Oral Medicine and Radiology, Bharati Vidyapeeth (Deemed to Be University) Dental College and Hospital, Pune, IND; 3 Department of Oral Pathology and Microbiology, Bharati Vidyapeeth (Deemed to Be University) Dental College and Hospital, Navi Mumbai, IND

**Keywords:** acid-etched implant surfaces, dental implants, dental pulp stem cells, photofunctionalization, sandblasting, titanium, ultraviolet

## Abstract

Background: Titanium implant surface bioactivity plays an important role in early cellular events associated with osseointegration. Sandblasted and acid-etched (SLA) surfaces improve cell-surface interaction, but biological aging may reduce their activity over time. Ultraviolet (UV) photofunctionalization has been proposed as a method to improve titanium surface bioactivity and enhance cellular response.

Aim: This study aimed to evaluate and compare the viability, proliferation, osteogenic differentiation, mineralization, and osteogenic gene expression of dental pulp stem cells (DPSCs) cultured on UV-pretreated and UV-untreated SLA titanium discs.

Methods: Human DPSCs were isolated from extracted third molars and characterized by flow cytometry. Three groups were evaluated: control, UV-untreated SLA titanium discs, and UV-treated SLA titanium discs. Cell viability was assessed using the MTT assay, proliferation by cell counting, morphology by confocal microscopy and scanning electron microscopy, osteogenic differentiation by RUNX2 expression and alkaline phosphatase (ALP) activity, mineralization by Alizarin Red S staining, and gene expression by quantitative real-time polymerase chain reaction for RUNX2, osteocalcin (OCN), and osteopontin (OPN).

Results: UV-treated SLA titanium showed significantly higher viability at 72 hours than UV-untreated SLA titanium and control groups, with values of 98.11% ± 0.78%, 96.11% ± 0.92%, and 94.22% ± 1.20%, respectively. Day 7 proliferation was also highest in the UV-treated group. RUNX2 expression, ALP activity, mineralization, and RUNX2, osteocalcin, and OPN gene expression were significantly greater in the UV-treated group, with all intergroup comparisons showing p < 0.001.

Conclusion: UV photofunctionalization enhanced the in vitro biological and osteogenic response of DPSCs on SLA titanium surfaces.

## Introduction

Dental implants have become a predictable treatment option for replacing missing teeth, largely because titanium surfaces can support osseointegration. However, the early biological response at the implant-bone interface depends strongly on surface characteristics such as roughness, surface energy, wettability, and surface chemistry. Sandblasted and acid-etched (SLA) titanium surfaces are widely used because their microrough topography improves early bone apposition and cellular interaction compared with smoother implant surfaces [1].

Despite these advances, titanium surfaces are not biologically static. During storage and handling, they undergo “biological aging,” mainly due to hydrocarbon accumulation, which reduces hydrophilicity and surface bioactivity. Ultraviolet (UV) photofunctionalization has been proposed as a practical method to reverse this aging by reducing surface carbon contamination, improving hydrophilicity, and enhancing protein adsorption and cellular attachment [2]. Experimental evidence has also shown that UV-treated titanium can promote mesenchymal stem cell migration, attachment, proliferation, and osteogenic differentiation, particularly on acid-etched titanium surfaces [3]. These early cellular events are clinically relevant because they form the biological basis for bone formation at the implant surface and may influence the early stages of osseointegration.

Dental pulp stem cells (DPSCs) are a useful cell model for evaluating implant surface bioactivity because they are accessible from extracted teeth, show high proliferative capacity, and possess osteogenic differentiation potential [4,5]. In the present study, DPSCs were not used as a clinical surface treatment for implants; rather, they were used as a biologically relevant mesenchymal stem cell model to assess how UV-treated and untreated SLA titanium surfaces influence cellular viability, proliferation, and osteogenic behavior. Previous studies have evaluated DPSC interactions with implant-related biomaterials and have suggested that treated titanium surfaces may enhance DPSC attachment and osteogenic activity [6,7].

However, limited evidence is available regarding the combined assessment of cytotoxicity, proliferation, cell morphology, mineralization, early osteogenic differentiation, and osteogenic gene expression of DPSCs on UV-photofunctionalized SLA titanium surfaces. Therefore, the primary objective of this in vitro study was to evaluate the effect of UV photofunctionalization on DPSC behavior on SLA titanium discs compared with UV-untreated SLA titanium and control conditions. The secondary objectives were to isolate and characterize DPSCs, assess cytotoxicity and proliferation, evaluate cell morphology and initial osteogenic differentiation, quantify mineralization, and compare the expression of osteogenic markers RUNX2, osteocalcin (OCN), and osteopontin (OPN) among the study groups. The study was designed to assess short-term cellular and molecular responses relevant to early osseointegration, without directly evaluating clinical implant success.

## Materials and methods

Study design

This in vitro experimental study was conducted in the Department of Periodontology from July 2024 to August 2025. The study protocol was approved by the institutional ethical review board (Reference Letter No. BVDU/IEC/R4/09/2023-24, dated May 15, 2025). Three experimental groups were evaluated: Group I: DPSCs cultured without titanium discs as the control group; Group II: DPSCs cultured on UV-untreated SLA titanium discs; and Group III: DPSCs cultured on UV-pretreated SLA titanium discs. The study assessed cell viability, proliferation, attachment, morphology, osteogenic differentiation, mineralization, and osteogenic gene expression. Each group included nine experimental replicates, arranged as three independent experimental runs with triplicate measurements per group.

Materials

Commercially manufactured SLA titanium discs were used as the test substrate. The discs were obtained from I-Fix, Faridabad and New Delhi, India. All discs were made of Grade 5 extra low interstitial titanium and measured 8 mm in diameter and 1 mm in thickness. A ultraviolet C (UV-C) germicidal lamp with a wavelength of 254 nm was used for photofunctionalization. Extracted human third molars were used to isolate DPSCs. The cell culture materials included Dulbecco’s Modified Eagle Medium (DMEM), fetal bovine serum (FBS), phosphate-buffered saline (PBS), penicillin-streptomycin solution, antibiotic-antimycotic solution, and 0.25% Trypsin-EDTA. Enzymatic digestion of pulp tissue was carried out using collagenase type I and dispase. The assay reagents included MTT reagent, dimethyl sulfoxide (DMSO), Calcein-AM, 4′,6-diamidino-2-phenylindole (DAPI), anti-RUNX2 antibody, Alizarin Red S staining solution, alkaline phosphatase (ALP) assay kit, RNA extraction reagent, RNA-to-complementary DNA (cDNA) conversion kit, SYBR Green Master Mix, and primers for RUNX2, OCN, OPN, and housekeeping genes.

Sample size and replication strategy

The sample size was determined based on the replication strategy commonly used in comparable in vitro cell-biomaterial experiments and on the need to obtain reproducible results across independent experimental runs. Previous in vitro work on UV-photofunctionalized titanium has demonstrated measurable differences in stem cell attachment, proliferation, and osteogenic differentiation between UV-treated and untreated titanium surfaces [3]. Therefore, each group was assessed using nine experimental replicates, arranged as three independent experimental runs with triplicate measurements per group. This approach also allowed assessment of consistency across repeated laboratory runs and minimized the influence of isolated technical variation [8].

Titanium disc surface preparation

The titanium discs were commercially processed to obtain an SLA surface. Initial meta-cleaning was performed using the MetaClean MS solution (Sharma Chemindus Pvt Ltd, India) at 45 kHz and 60°C for 30 minutes. Sandblasting was then carried out using 300 µm alumina particles. The alumina particles were heated to 400°C before use, and each disc was sandblasted for 10 seconds at a gun pressure of 4.5 bar. Precleaning was performed using MetaClean MS and purified water at 60°C in three ultrasonic stages: 25 kHz for 30 minutes, 45 kHz for 30 minutes, and 132 kHz for 30 minutes.

Acid etching was performed using a triple-acid etching protocol. The discs were treated with hydrochloric acid at 80°C for seven minutes in a silicon oil water bath, followed by sulfuric acid at 110°C for seven minutes in a silicon oil water bath, and nitric acid at 25°C for 30 minutes. Aqueous cleaning was then carried out using purified type II water with a pH between 5 and 7 and total dissolved solids not exceeding 500 mg/L. This was performed in four stages using different ultrasonic frequencies. The discs were finally dried under compression at 60°C-65°C for 10 minutes. After surface preparation, the discs were handled aseptically throughout the laboratory procedures to minimize contamination.

UV photofunctionalization protocol

The discs allocated to the UV-treated group were exposed to UV-C irradiation using a 254 nm germicidal lamp (Philips TUV 30 W, The Netherlands). The titanium discs were placed at a fixed distance of 10 cm from the UV source and exposed to an irradiance intensity of approximately 2.0 mW/cm² for 24 hours under sterile conditions. During irradiation, the discs were placed in sterile, uncovered Petri dishes inside a laminar airflow chamber to minimize contamination and to allow uniform exposure of the treated surface.

After UV treatment, the discs were immediately transferred aseptically into sterile culture plates and conditioned in 3 mL of phenol red-free DMEM supplemented with glutamine and antibiotic-antimycotic solution for two hours at 37°C before cell seeding. This step was performed to allow surface equilibration before contact with cells. UV-untreated SLA titanium discs underwent identical handling and conditioning procedures, except that they were not exposed to UV-C irradiation.

Surface physicochemical characterization

All titanium discs used in this study were commercially manufactured and processed using a standardized SLA surface-treatment protocol. Additional physicochemical characterization, including contact angle measurements, X-ray photoelectron spectroscopy, atomic force microscopy, and quantitative surface roughness evaluation, was not performed in the present study because the primary focus was on evaluating the biological response of DPSCs to UV-treated and UV-untreated SLA titanium surfaces. The absence of direct surface characterization data was considered a methodological limitation and has been acknowledged in the Discussion section.

Collection of dental pulp tissue

Healthy impacted third molars extracted for orthodontic or therapeutic reasons were used for DPSC isolation. The teeth were obtained from patients aged 14-28 years after written informed consent. This age range was selected because impacted third molars are commonly extracted in adolescents and young adults, making them an accessible and ethically acceptable source of dental pulp tissue. In addition, dental pulp tissue from younger permanent teeth generally contains a higher proportion of viable and proliferative mesenchymal stem cells with better regenerative and osteogenic potential than pulp tissue from older individuals. Restricting the donor age range also helped reduce biological variability in DPSC behavior across experimental samples.

Before extraction, each participant was instructed to rinse with chlorhexidine gluconate mouthwash to reduce microbial contamination. The extraction procedure was performed under sterile conditions. After extraction, the tooth surface was disinfected, and the pulp chamber was accessed at the cementoenamel junction using a sterile fissure bur. The pulp tissue was carefully extirpated using sterile barbed broaches and immediately transferred into PBS supplemented with double-strength antibiotic-antimycotic solution.

Isolation and culture of DPSCs

The retrieved pulp tissue was minced into approximately 1 mm fragments and transferred into an enzymatic digestion solution containing collagenase type I and dispase, prepared in sterile PBS with antibiotics. The tissue was incubated at 37°C for 60 minutes, with intermittent vortexing at 30 minute intervals to facilitate tissue dissociation. The resulting cell suspension was filtered through a 70 μm cell strainer to obtain a single-cell suspension. Enzymatic activity was neutralized using Minimum Essential Medium supplemented with 10% FBS, and the suspension was centrifuged at 67 × g for five minutes at room temperature.

The cell pellet was resuspended in complete culture medium consisting of DMEM supplemented with 20% FBS and antibiotic-antimycotic solution. Cells were seeded into T25 culture flasks and incubated at 37°C in a humidified incubator containing 5% CO₂ and 95% atmospheric air. The culture medium was replaced every two to three days, and cellular outgrowth was monitored using an inverted phase-contrast microscope. Once the cells reached 70%-80% confluence, they were detached using 0.25% Trypsin-ethylenediaminetetraacetic acid (EDTA) and subcultured into T75 flasks. Cells from passage 2 onward were used for experimental procedures. Cell seeding density was standardized according to the specific assay protocol.

Characterization of DPSCs

DPSCs at passage 3 were characterized by flow cytometry to confirm their mesenchymal stem cell phenotype. The cells were stained for mesenchymal stem cell-positive markers CD73, CD90, and CD105 and were simultaneously evaluated for the absence of hematopoietic markers CD34 and CD45. The results were expressed as percentage positivity for each marker. This immunophenotypic profile was used to confirm that the isolated cells fulfilled the expected mesenchymal stem cell characteristics before their use in downstream experimental assays.

Cytotoxicity and cell viability assessment

Cell viability and cytotoxicity were assessed using the MTT colorimetric assay. Conditioned media were prepared by incubating the respective titanium discs in 3 mL of phenol red-free DMEM supplemented with antibiotics, glutamine, and FBS at 37°C for 72 hours. DPSCs were trypsinized and seeded into 96-well culture plates at a density of 1 × 10⁴ cells per well. The cells were cultured in the respective conditioned media for 24, 48, and 72 hours.

At each time interval, 50 μL of MTT solution was added to each well and incubated for four hours at 37°C. The formazan crystals formed by metabolically active cells were dissolved in DMSO, and absorbance was measured spectrophotometrically at 570 nm. Cell viability was calculated and expressed as a percentage relative to the control group.

Cell proliferation, attachment, and confocal microscopy

For evaluation of proliferation and attachment, DPSCs were seeded on SLA titanium discs placed in confocal culture dishes at an initial density of 20,000 cells per dish. A control group of DPSCs cultured without titanium discs was maintained for baseline comparison. Cell counts were recorded on alternate days over a nine-day period to assess proliferation.

On days 3 and 7, cell viability and nuclear morphology were assessed using dual-fluorescence staining. Calcein-AM was used to stain live cells, while DAPI was used for nuclear staining. After staining, the cells were washed three times with PBS to remove excess dye. The samples were examined using confocal laser scanning microscopy to assess cell adhesion, distribution, viability, and nuclear integrity across the experimental groups.

Scanning electron microscopy

Cell morphology and cell-surface interaction were assessed using scanning electron microscopy after seven days of culture. DPSCs were seeded on titanium discs at a density of 20,000 cells per disc. At the selected time point, the cell-disc constructs were gently washed twice with PBS to remove nonadherent cells and residual medium. The samples were fixed with 2.5% glutaraldehyde for four hours at room temperature and dehydrated through graded ethanol concentrations.

After complete dehydration, the specimens were dried at 37°C and sputter-coated with a thin layer of gold-palladium to improve electrical conductivity. The prepared specimens were examined under a scanning electron microscope (XL30 FEG; FEI, Eindhoven, The Netherlands) at an accelerating voltage of 10 kV to compare cell spreading, attachment, and morphology on UV-treated and UV-untreated SLA titanium surfaces.

Intracellular RUNX2 expression

Intracellular RUNX2 expression was evaluated on days 7 and 21 after seeding using fluorescence-activated cell sorting. At each time point, adherent cells were washed with PBS, harvested, fixed, and permeabilized. The cells were incubated with anti-RUNX2 antibody at 4°C for 30 minutes, followed by repeated PBS washes to remove unbound antibody. Quantitative analysis was performed using a FACSCalibur system (BD Biosciences, BD Biosciences, CA), and intracellular RUNX2 expression was expressed as mean fluorescence intensity. The results were compared among the control, UV-untreated SLA, and UV-treated SLA groups.

Alizarin Red S staining

Extracellular matrix mineralization was assessed after 21 days of culture using Alizarin Red S staining. At the designated time point, adherent cells were washed with PBS and fixed in 4% paraformaldehyde for 15 minutes at room temperature. The specimens were rinsed with deionized water, followed by application of 1% Alizarin Red S solution for 20 minutes. Excess stain was removed by repeated washing with deionized water.

For quantitative assessment, the bound stain was extracted, and absorbance was measured at 562 nm. Stained samples were also examined under light microscopy at 10× magnification to qualitatively assess the extent of calcium deposition and mineralized matrix formation among the groups.

ALP activity assay

ALP activity was assessed on days 7 and 14 as an indicator of early osteogenic differentiation. ALP activity was measured using a commercially available colorimetric assay kit with p-nitrophenyl phosphate as the enzymatic substrate, and absorbance was read at 405 nm. Total protein concentration was determined using a micro-bicinchoninic acid assay to normalize ALP activity. The final ALP activity values were expressed as optical density per milligram of total protein.

For qualitative assessment, cells were washed twice with PBS, fixed for five minutes at room temperature, and incubated with ALP substrate solution for 15 minutes. ALP-positive cells, identified by purple staining, were visualized under optical microscopy.

qRT-PCR analysis

Osteogenic gene expression was assessed using quantitative real-time polymerase chain reaction on days 7 and 14 after seeding. Total RNA was extracted from cells adherent to the titanium discs using a commercially available RNA extraction reagent. The extracted RNA was purified, including DNase treatment to remove genomic DNA contamination. RNA quality and concentration were assessed using a Nanodrop spectrophotometer.

One microgram of total RNA was reverse-transcribed into cDNA using an RNA-to-cDNA synthesis kit. Quantitative real-time polymerase chain reaction (qRT-PCR) was performed using SYBR Green Master Mix and specific primers for RUNX2, OCN, and OPN. Gene expression levels were normalized to the housekeeping gene. Relative gene expression was calculated using the 2^-ΔΔCt^ method and expressed as fold change relative to the control group.

Statistical analysis

All quantitative data were expressed as mean ± standard deviation. Statistical analysis was performed using Statistical Package for the Social Sciences software, version 29.0 (IBM Corp., Armonk, NY). The Shapiro-Wilk test was used to assess the normality of data distribution. For normally distributed data, intergroup comparisons among the three study groups were performed using one-way analysis of variance (ANOVA). When a significant difference was identified on ANOVA, post hoc pairwise comparisons were performed using Tukey’s honestly significant difference (HSD) test. For outcomes measured at more than one time point, comparisons were performed separately at each time point. F-statistics and corresponding p values were reported for ANOVA results, while mean differences and p values were reported for Tukey’s HSD post hoc comparisons. A p value of ≤0.05 was considered statistically significant. Post hoc pairwise comparisons were performed using Tukey’s HSD following one-way ANOVA, as reflected in the post hoc table.

## Results

Characterization of DPSCs

Flow cytometric analysis confirmed that the isolated cells showed a mesenchymal stem cell phenotype. The cells demonstrated high expression of CD73, CD90, and CD105, with all three markers showing mean positivity above 95%. In contrast, the hematopoietic markers CD34 and CD45 showed minimal expression, with mean values below 2%. The detailed marker expression profile is presented in Table 1.

**Table 1:** Flow cytometric characterization of dental pulp stem cells Values are presented as mean ± standard deviation. No inferential statistical test was applied, as these data represent descriptive immunophenotypic characterization

Marker	Marker type	Mean expression (%)	Standard deviation
CD73	Mesenchymal stem cell marker	97.25	1.85
CD90		96.40	2.10
CD105		95.88	2.35
CD34	Hematopoietic marker	1.85	0.92
CD45		1.12	0.68

Cell viability and proliferation

Cell viability increased progressively from 24 to 72 hours in all groups. At 72 hours, the highest viability was observed in the UV-treated SLA titanium group, followed by the UV-untreated SLA titanium group and the control group, with mean values of 98.11% ± 0.78%, 96.11% ± 0.92%, and 94.22% ± 1.20%, respectively. Intergroup differences were statistically significant at 24, 48, and 72 hours, with p < 0.001 at each interval.

**Figure 1:**
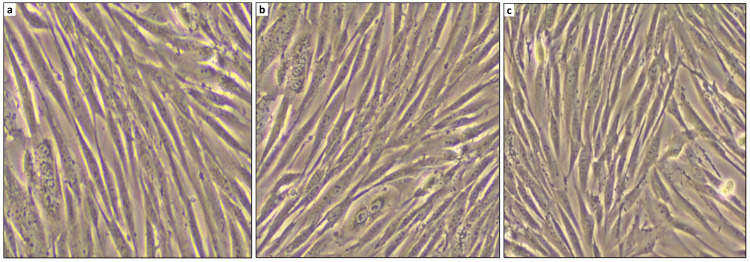
Scanning electron microscopy showing images of dental pulp stem cell morphology and surface attachment. (a) Control group. (b) UV-untreated group. (c) UV-pretreated group UV: ultraviolet

Cell proliferation also increased from days 3 to 7 in all groups. On day 7, the UV-treated SLA titanium group showed the highest proliferation count, compared with the UV-untreated SLA titanium group and the control group (Figure 1). Intergroup differences were statistically significant on both days 3 and 7 (p < 0.001). The detailed values are shown in Table 2. Tukey’s HSD post hoc analysis showed significant pairwise differences among the control, UV-untreated SLA titanium, and UV-treated SLA titanium groups for cell viability at 24, 48, and 72 hours and for cell proliferation at days 3 and 7, with all pairwise comparisons showing p < 0.001.

**Table 2:** Comparison of cell viability and proliferation among the study groups One-way ANOVA analysis was performed SLA: sandblasted and acid-etched; UV: ultraviolet; ANOVA: analysis of variance

Outcome	Time point	Control	UV-untreated SLA titanium	UV-treated SLA titanium	Test value	p value
Cell viability (%)	24 hours	88.44 ± 1.13	90.88 ± 1.05	93.33 ± 1.00	F(2,24) = 47.76	<0.001
	48 hours	91.33 ± 1.00	93.88 ± 0.95	96.44 ± 0.88	F(2,24) = 65.84	<0.001
	72 hours	94.22 ± 1.20	96.11 ± 0.92	98.11 ± 0.78	F(2,24) = 35.29	<0.001
Cell proliferation	Day 3	21,277.78 ± 501.94	23,888.89 ± 520.10	26,288.89 ± 537.23	F(2,24) = 209.11	<0.001
	Day 7	28,311.11 ± 491.03	32,111.11 ± 560.45	36,455.56 ± 612.60	F(2,24) = 481.90	<0.001

RUNX2 expression, ALP activity, and mineralization

RUNX2 expression increased from day 7 to 21 in all groups. At day 21, the UV-treated SLA titanium group showed the highest RUNX2 expression, 2.05 ± 0.03, compared with 1.66 ± 0.03 in the UV-untreated SLA titanium group and 1.27 ± 0.02 in the control group. Intergroup differences were statistically significant on both days 7 and 21 (p < 0.001).

**Figure 2:**
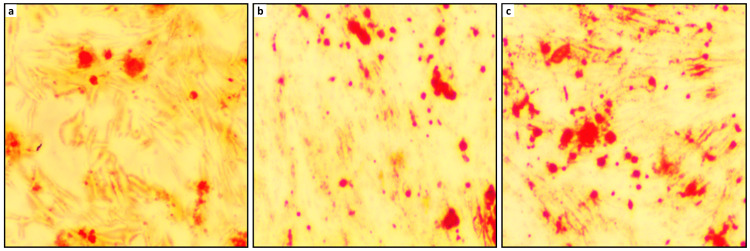
Increasing mineralization activity. (a) Control group. (b) UV-untreated group. (c) UV-pretreated group Alizarin Red S staining has been done UV: ultraviolet

ALP activity showed a similar pattern. On day 14, ALP activity was highest in the UV-treated SLA titanium group, 2.42 ± 0.03, followed by the UV-untreated SLA titanium group, 1.89 ± 0.03, and the control group, 1.36 ± 0.02. Mineralization at day 21 was also highest in the UV-treated group (Figure 2), with an OD562 value of 0.71 ± 0.02. All intergroup comparisons for RUNX2 expression, ALP activity, and mineralization were statistically significant, with p < 0.001. These findings are summarized in Table 3. Tukey’s HSD post hoc comparisons also showed significant differences between each pair of groups for RUNX2 expression at day 21, ALP activity at day 14, and mineralization at day 21, with p < 0.001 for all pairwise comparisons (see the Appendix).

**Table 3:** Comparison of osteogenic differentiation markers among the study groups One-way ANOVA analysis was performed SLA: sandblasted and acid-etched; UV: ultraviolet; RUNX2: runt-related transcription factor 2; MFI: mean fluorescence intensity; ALP: alkaline phosphatase; OD562: optical density measured at 562 nm; ANOVA: analysis of variance

Outcome	Time point	Control	UV-untreated SLA titanium	UV-treated SLA titanium	Test value	p value
RUNX2 expression, MFI	Day 7	1.15 ± 0.02	1.48 ± 0.03	1.80 ± 0.03	F(2,24) = 1,296.41	<0.001
	Day 21	1.27 ± 0.02	1.66 ± 0.03	2.05 ± 0.03	F(2,24) = 1,866.68	<0.001
ALP activity	Day 7	1.08 ± 0.02	1.58 ± 0.03	2.07 ± 0.04	F(2,24) = 2,281.34	<0.001
	Day 14	1.36 ± 0.02	1.89 ± 0.03	2.42 ± 0.03	F(2,24) = 3,447.41	<0.001
Mineralization, OD562	Day 21	0.33 ± 0.02	0.52 ± 0.02	0.71 ± 0.02	F(2,24) = 812.25	<0.001

Osteogenic gene expression

qRT-PCR analysis showed statistically significant intergroup differences in RUNX2, OCN, and OPN expression, with p < 0.001 for all three genes. RUNX2 expression was highest in the UV-treated SLA titanium group, 1.95 ± 0.05, compared with 1.52 ± 0.04 in the UV-untreated SLA titanium group and 1.10 ± 0.02 in the control group. Similar patterns were observed for OCN and OPN expression, with the UV-treated group showing the highest relative gene expression values. Post hoc analysis confirmed significant pairwise differences among all three groups for RUNX2, OCN, and OPN gene expression, with p < 0.001 for each comparison. The detailed gene expression data are presented in Table 4.

**Table 4:** Relative osteogenic gene expression among the study groups One-way ANOVA analysis was performed SLA: sandblasted and acid-etched; UV: ultraviolet; RUNX2: runt-related transcription factor 2; ANOVA: analysis of variance

Gene	Control	UV-untreated SLA titanium	UV-treated SLA titanium	Test value	p value
RUNX2	1.10 ± 0.02	1.52 ± 0.04	1.95 ± 0.05	F(2,24) = 1,083.80	<0.001
Osteocalcin	1.21 ± 0.03	1.68 ± 0.04	2.16 ± 0.05	F(2,24) = 1,218.42	<0.001
Osteopontin	1.31 ± 0.03	1.83 ± 0.04	2.35 ± 0.05	F(2,24) = 1,460.16	<0.001

## Discussion

The present in vitro study demonstrated that UV photofunctionalization of SLA titanium surfaces enhanced the biological response of DPSCs across multiple parameters, including cell viability, proliferation, RUNX2 expression, ALP activity, matrix mineralization, and osteogenic gene expression. The UV-treated SLA titanium group consistently showed higher values than the UV-untreated SLA titanium and control groups, indicating that UV treatment produced a broader pro-osteogenic cellular response rather than an isolated improvement in a single assay. This pattern is biologically relevant because early cell attachment, proliferation, matrix maturation, and mineral deposition represent sequential events in osteoblastic differentiation and bone formation at the implant-tissue interface [9].

The improved cell viability and proliferation observed in the UV-treated group may be related to physicochemical changes induced by UV irradiation. UV photofunctionalization has been reported to reduce hydrocarbon contamination, improve hydrophilicity, and increase titanium surface bioactivity, thereby favoring protein adsorption and early cellular attachment [2,10]. Aita et al. showed that UV-treated acid-etched titanium promoted human mesenchymal stem cell migration, attachment, proliferation, and osteogenic differentiation, which supports the present findings in a stem-cell-based experimental model [3]. The importance of surface energy in improving osteoblast response to titanium has also been demonstrated previously, suggesting that surface activation may influence early cell-surface interactions independently of surface roughness alone [11].

Although the differences in cell viability were statistically significant, the absolute increase was modest. At 72 hours, viability was 98.11% ± 0.78% in the UV-treated SLA titanium group compared with 96.11% ± 0.92% in the UV-untreated SLA titanium group and 94.22% ± 1.20% in the control group. Therefore, the viability data should be interpreted as evidence of favorable cellular compatibility rather than as a large, standalone biological effect. The greater biological relevance is evidenced by the parallel improvements in proliferation, osteogenic marker expression, ALP activity, and mineralization, which together indicate a more consistent osteogenic response on UV-treated SLA titanium surfaces.

The findings also align with previous studies that emphasize the role of wettability and surface energy in early osseointegration. Buser et al. reported greater early bone apposition around chemically modified hydrophilic SLA surfaces compared with conventional SLA surfaces [1]. Similarly, Sawase et al. observed that photoinduced hydrophilicity enhanced initial cell behavior and early bone apposition [12]. These observations support the biological plausibility that UV-treated SLA titanium may create a more favorable surface environment for DPSC attachment, spreading, and downstream osteogenic activity [13]. In the present study, SEM and confocal observations supported improved cell attachment and morphology on treated surfaces, although these imaging findings were descriptive and were not evaluated using blinded quantitative image analysis.

The increase in RUNX2 expression, ALP activity, and Alizarin Red mineralization provides evidence of enhanced osteogenic differentiation. RUNX2 is a key transcription factor involved in the commitment of mesenchymal cells toward the osteoblastic lineage [14]. ALP reflects early mineralization-associated activity, while Alizarin Red staining indicates calcium-rich mineralized matrix formation during later stages of differentiation [15]. The higher expression of RUNX2, OCN, and OPN in the UV-treated group further supports enhanced osteogenic commitment at the molecular level. These results are consistent with Roy et al., who reported that UV-C-treated titanium oxide surfaces improved survival and osteogenic differentiation of precursor cells, including increased osteogenesis-related gene expression [16].

The use of DPSCs adds relevance to the present experimental model because these cells are accessible from extracted teeth and have recognized proliferative and osteogenic potential. Gronthos et al. first described postnatal human DPSCs as a clonogenic and highly proliferative cell population capable of forming mineralized tissue [4]. Other studies have also shown that dental pulp-derived cells can contribute to osteogenic and bone-forming responses, supporting their use in implant-surface research [17,18]. In the present study, DPSCs were used as a biological model to test the cellular response to titanium surface modification; they were not used as a clinical coating or treatment applied to implants. Therefore, issues such as clinical storage of implants after DPSC application were not part of this experimental design. More directly, Guo et al. demonstrated improved DPSC attachment and osteogenic potential on UV-treated titanium and other implant-related biomaterials, findings that closely support the present findings [6].

The clinical significance of these findings lies in the potential role of UV photofunctionalization as a chairside surface-activation approach before implant placement. Since early cell attachment, proliferation, osteogenic differentiation, and mineralization are important biological events during osseointegration, the enhanced DPSC response observed in this study suggests that UV-treated SLA titanium may provide a more biologically favorable surface for early bone-forming activity. This may be relevant in clinical situations where improved early bone response is desirable, such as immediate implant placement, sites with compromised bone quality, or patients with delayed healing potential. However, these implications must be interpreted cautiously because the present study provides in vitro biological evidence only. Animal studies and clinical trials are required to confirm whether these cellular advantages translate into improved implant stability, faster osseointegration, or reduced early implant failure [19,20].

The present study has several limitations. First, the in vitro design cannot reproduce the full biological complexity of the peri-implant environment, including vascularity, immune response, mechanical loading, and bone remodeling. Second, although standardized commercially manufactured SLA titanium discs were used, direct physicochemical characterization by contact-angle analysis, X-ray photoelectron spectroscopy, atomic force microscopy, or quantitative roughness testing was not performed. Therefore, the mechanism was inferred from established literature rather than directly measured in the present experiment. Third, the 24-hour UV exposure protocol may differ from shorter chairside photofunctionalization protocols used clinically. Fourth, donor-specific details such as exact donor number, sex distribution, and mean age were not available for subgroup analysis, and donor-level variability could not be fully assessed. The distinction between experimental replicates and biological donor variability remains important in cell-culture research [8]. Finally, formal blinding during SEM and confocal image assessment was not performed, and the final terminal sterilization protocol after commercial SLA processing was not independently verified. Future studies should include detailed surface characterization, donor-matched biological replicates, shorter clinically practical UV protocols, quantitative image analysis, three-dimensional or co-culture models, and animal or clinical validation before definitive clinical recommendations are made.

## Conclusions

Within the limitations of this in vitro study, UV photofunctionalization of SLA titanium discs enhanced DPSC viability, proliferation, osteogenic differentiation, mineralization, and osteogenic gene expression compared with UV-untreated titanium surfaces. These findings indicate that UV-treated SLA titanium may provide a more favorable surface for early cellular and osteogenic responses relevant to osseointegration. However, direct clinical benefits cannot be inferred from these results alone, and further surface-characterization studies, animal experiments, and clinical trials are required before recommending this approach for routine implant practice.

## Appendices

**Table 5:** Tukey’s HSD post hoc pairwise comparisons among study groups SLA: sandblasted and acid-etched; UV: ultraviolet; RUNX2: runt-related transcription factor 2; ALP: alkaline phosphatase; OD562: optical density measured at 562 nm; HSD: honestly significant difference

Outcome	Time point	Group comparison	Mean difference	p value
Cell viability (%)	24 hours	Control vs. UV-untreated SLA	-2.44	<0.001
		Control vs. UV-treated SLA	-4.89	<0.001
		UV-untreated SLA vs. UV-treated SLA	-2.45	<0.001
	48 hours	Control vs. UV-untreated SLA	-2.55	<0.001
		Control vs. UV-treated SLA	-5.11	<0.001
		UV-untreated SLA vs. UV-treated SLA	-2.56	<0.001
	72 hours	Control vs. UV-untreated SLA	-1.89	<0.001
		Control vs. UV-treated SLA	-3.89	<0.001
		UV-untreated SLA vs. UV-treated SLA	-2.00	<0.001
Cell proliferation	Day 3	Control vs. UV-untreated SLA	-2,611.11	<0.001
		Control vs. UV-treated SLA	-5,011.11	<0.001
		UV-untreated SLA vs. UV-treated SLA	-2,400.00	<0.001
	Day 7	Control vs. UV-untreated SLA	-3,800.00	<0.001
		Control vs. UV-treated SLA	-8,144.45	<0.001
		UV-untreated SLA vs. UV-treated SLA	-4,344.45	<0.001
RUNX2 expression	Day 21	Control vs. UV-untreated SLA	-0.39	<0.001
		Control vs. UV-treated SLA	-0.78	<0.001
		UV-untreated SLA vs. UV-treated SLA	-0.39	<0.001
ALP activity	Day 14	Control vs. UV-untreated SLA	-0.53	<0.001
		Control vs. UV-treated SLA	-1.06	<0.001
		UV-untreated SLA vs. UV-treated SLA	-0.53	<0.001
Mineralization (OD562)	Day 21	Control vs. UV-untreated SLA	-0.19	<0.001
		Control vs. UV-treated SLA	-0.38	<0.001
		UV-untreated SLA vs. UV-treated SLA	-0.19	<0.001
RUNX2 gene expression	-	Control vs. UV-untreated SLA	-0.42	<0.001
		Control vs. UV-treated SLA	-0.85	<0.001
		UV-untreated SLA vs. UV-treated SLA	-0.43	<0.001
Osteocalcin	-	Control vs. UV-untreated SLA	-0.47	<0.001
		Control vs. UV-treated SLA	-0.95	<0.001
		UV-untreated SLA vs. UV-treated SLA	-0.48	<0.001
Osteopontin	-	Control vs. UV-untreated SLA	-0.52	<0.001
		Control vs. UV-treated SLA	-1.04	<0.001
		UV-untreated SLA vs. UV-treated SLA	-0.52	<0.001
